# The safety/tolerability of opicapone when used early in Parkinson's disease patients with levodopa-induced motor fluctuations: A *post-hoc* analysis of BIPARK-I and II

**DOI:** 10.3389/fneur.2022.994114

**Published:** 2022-08-23

**Authors:** José-Francisco Rocha, Georg Ebersbach, Andrew Lees, Eduardo Tolosa, Joaquim J. Ferreira, Werner Poewe, Olivier Rascol, Fabrizio Stocchi, Angelo Antonini, Diogo Magalhães, Helena Gama, Patrício Soares-da-Silva

**Affiliations:** ^1^BIAL-Portela & C^a^ S.A., Coronado, Portugal; ^2^Movement Disorders Clinic, Beelitz-Heilstätten, Germany; ^3^National Hospital for Neurology and Neurosurgery, London, United Kingdom; ^4^Parkinson Disease and Movement Disorder Unit, Neurology Service, Hospital Clínic de Barcelona, Institut d'Investigacions Biomédiques August Pi i Sunyer (IDIBAPS), University of Barcelona (UB), Centro de Investigación Biomédica en Red sobre Enfermedades Neurodegenerativas (CIBERNED), Barcelona, Spain; ^5^Laboratory of Clinical Pharmacology and Therapeutics, Faculty of Medicine, University of Lisbon, Lisbon, Portugal; ^6^Department of Neurology, Medical University of Innsbruck, Innsbruck, Austria; ^7^Toulouse Parkinson Expert Center, Departments of Neurosciences and Clinical Pharmacology, Centre d'Investigation Clinique de Toulouse CIC1436, NS-Park/FCRIN Network, and NeuroToul COEN Center, University Hospital of Toulouse, INSERM, University of Toulouse 3, Toulouse, France; ^8^Department of Neurology, IRCCS San Raffaele Pisana, Rome, Italy; ^9^Parkinson and Movement Disorders Unit, Center for Neurodegenerative Disease (CESNE), Department of Neurosciences, University of Padova, Padova, Italy

**Keywords:** catechol-O-methyltransferase inhibitor, levodopa, motor fluctuations, opicapone, Parkinson's disease, safety/tolerability, wearing off

## Abstract

**Introduction:**

*Post-hoc* analyses of the BIPARK-I and II trials previously demonstrated that opicapone (OPC) 50 mg was efficacious over the whole trajectory of motor fluctuation evolution in patients with Parkinson's disease (PD) and end-of-dose motor fluctuations, with enhanced efficacy in patients who were earlier vs. later in their disease course and levodopa treatment pathway. Complementary *post-hoc* analyses were performed to evaluate the safety/tolerability of OPC following the same pre-defined segmentation of the wide spectrum of duration of both PD and levodopa therapy, as well as of motor fluctuation history, in this patient population.

**Materials and methods:**

Data from matching treatment arms in BIPARK-I and II were combined for the placebo (PLC) and OPC 50 mg groups and exploratory *post-hoc* analyses were performed to investigate the safety/tolerability of OPC 50 mg and PLC in 22 subgroups of patients who were in “earlier” vs. “later” stages of both their disease course (e.g., duration of PD <6 years vs. ≥6 years) and levodopa treatment pathway (e.g., levodopa treatment duration <4 vs. ≥4 years). Safety/tolerability assessments included evaluation of treatment-emergent adverse events (TEAEs).

**Results:**

The Safety Set included 522 patients (PLC, *n* = 257; OPC 50 mg, *n* = 265). For OPC 50 mg, incidences of TEAEs, related TEAEs, related serious TEAEs, and related TEAEs leading to discontinuation were lower for patients in earlier vs. later stages of their disease course and levodopa treatment pathway in 86.4, 86.4, 63.6, and 68.2% of the 22 pairwise comparisons conducted, respectively (compared with 63.6, 77.3, 18.2, and 45.5%, respectively, in the 22 corresponding PLC comparisons).

**Conclusion:**

OPC 50 mg was generally well-tolerated when used to treat patients with PD with end-of-dose fluctuations, with an even more favorable tolerability profile in patients who were earlier, as opposed to later, in their disease course and levodopa treatment pathway, further supporting its use as an early adjunct to levodopa in PD.

## Introduction

Levodopa (L-DOPA) is the most efficacious pharmacological treatment for Parkinson's disease (PD), but its benefit is compromised in many patients by the development of motor fluctuations and dyskinesias (1–3). It is thought that response fluctuations and drug-induced dyskinesias emerge during sustained L-DOPA treatment due to pulsatile stimulation of striatal dopamine receptors following intermittent exogenous drug delivery (as opposed to continuous physiological stimulation of the receptors) (4–6). This results in downstream changes in the basal ganglia, which is exacerbated over time by the continuing death of nigrostriatal neurons (4–6). Improvement in the bioavailability and steadiness of pharmacologically administered L-DOPA could extend ON-time and not only reduce motor complications in patients with motor fluctuations but also reduce the onset of motor fluctuations in those in early stages of PD, when the buffering capacity of surviving neurons is still relatively intact, and the priming effect is less profound than in patients with more advanced disease (7, 8). Within this context, co-administration of L-DOPA with a catechol-O-methyltransferase (COMT) inhibitor may facilitate a more stable delivery of L-DOPA to the brain by extending its half-life and bioavailability (9).

Opicapone (OPC) is a third-generation, once-daily COMT inhibitor developed to fulfill the need for a more potent, longer-acting COMT inhibitor (10–13). OPC has been shown to be generally well-tolerated and efficacious in reducing OFF-time in two pivotal trials in patients with PD and end-of-dose motor fluctuations (BIPARK-I and II) (14, 15). On the basis of these trials, OPC was first approved in the European Union as adjunctive therapy to preparations of L-DOPA/dopa decarboxylase inhibitors in adult patients with PD and end-of-dose motor fluctuations who cannot be stabilized on those combinations (16). It is currently also approved and marketed in the USA, Japan, South Korea, Australia and other countries.

We previously conducted exploratory *post-hoc* analyses of data from the BIPARK-I and II trials to evaluate the efficacy of OPC following a pre-defined segmentation of motor fluctuations in PD, based on baseline disease- and therapy-related characteristics (17). In this study, we have conducted additional *post-hoc* analyses to assess the safety/tolerability of OPC using the same approach.

## Materials and methods

### Study design

BIPARK-I and II were Phase III, multicenter, randomized, double-blind, placebo (PLC)-controlled trials of OPC as an adjunct to L-DOPA in patients with PD with end-of-dose motor fluctuations, the results of which have been published previously (14, 15). The trials had similar designs, eligibility criteria and methods (17). In BIPARK-I, patients were randomized to treatment with OPC (5, 25, or 50 mg once daily), PLC, or entacapone (200 mg with every L-DOPA intake) for 14–15 weeks (14). In BIPARK-II, patients were randomized to treatment with OPC (25 or 50 mg once daily) or PLC for 14–15 weeks (15). In both trials, the primary efficacy endpoint was change from baseline to endpoint in absolute OFF-time vs. PLC, based on patient diaries (14, 15).

The methodology employed in the current study has been described previously (17). Data from matching treatment arms in BIPARK-I and II were combined for the PLC and OPC 50 mg groups, and exploratory *post-hoc* analyses were performed to investigate the safety/tolerability of OPC 50 mg vs. PLC in patients who were divided on the basis of baseline disease- and therapy-related characteristics into representative subgroups of patients who were in “earlier” or “later” stages of both their disease course and L-DOPA treatment pathway.

### Study population

In BIPARK-I and II, eligible patients were male or female, aged 30–83 years, with a ≥3-year diagnosis of PD, Hoehn and Yahr 1–3 at ON-state, who were receiving L-DOPA treatment for ≥1 year and experiencing end-of-dose motor fluctuations. Details of the full inclusion/exclusion criteria from the trials have been published previously (14, 15). These *post-hoc* analyses included all patients treated with OPC 50 mg and PLC in BIPARK-I and II.

### Study assessments

Baseline characteristics and safety/tolerability were assessed for each patient pairwise baseline subgroup, defined on the basis of clinical variables reflecting different aspects of advancing PD: duration of PD (<6 years vs. ≥6 years; <7 years vs. ≥7 years; <8 years vs. ≥8 years; <9 years vs. ≥9 years); Hoehn and Yahr staging (<2.5 vs. ≥2.5); and timing of onset of motor fluctuations ( ≤ 1 year [termed “recent motor fluctuators”] vs. >1 year; ≤ 2 years [termed “early motor fluctuators”] vs. >2 years). Treatment-related characteristics consisted of: number of L-DOPA intakes (<4 vs. ≥4; <5 vs. ≥5; <6 vs. ≥6); L-DOPA treatment duration (<4 years vs. ≥4 years; <5 years vs. ≥5 years; <6 years vs. ≥6 years; <7 years vs. ≥7 years; <8 years vs. ≥8 years); L-DOPA daily amount (<500 mg vs. ≥500 mg; <600 mg vs. ≥600 mg; <700 mg vs. ≥700 mg; <800 mg vs. ≥800 mg); use of L-DOPA only (i.e., without a dopamine agonist [DA] or monoamine oxidase B inhibitor [MAO-BI]) (Yes vs. No); use of L-DOPA plus a DA (Yes vs. No); and use of L-DOPA plus a MAO-BI (Yes vs. No). Baseline characteristics were summarized for the above subgroups and included age, gender, absolute OFF-time, duration of PD, time since onset of motor fluctuations, Hoehn and Yahr staging at ON, L-DOPA daily dose, and duration of L-DOPA therapy.

Safety/tolerability assessments included the incidence of treatment-emergent adverse events (TEAEs), related TEAEs, related serious TEAEs, related TEAEs leading to discontinuation, and specific related dopaminergic-related TEAEs (dyskinesia, nausea, hallucination, orthostatic hypotension, and vomiting). Related TEAEs were defined as TEAEs for which the relationship to study drug was reported as “possible,” “probable,” “definite,” or missing.

### Statistical analyses

The assessments were conducted for the Safety Set, which included all patients who received at least one dose of study drug. Since this was an exploratory, descriptive *post-hoc* analysis, no formal statistical comparisons were conducted. Quantitative variables were described as mean with standard deviation (SD) and qualitative variables were described as percentage frequencies.

## Results

### Study population

**Figure 1:**
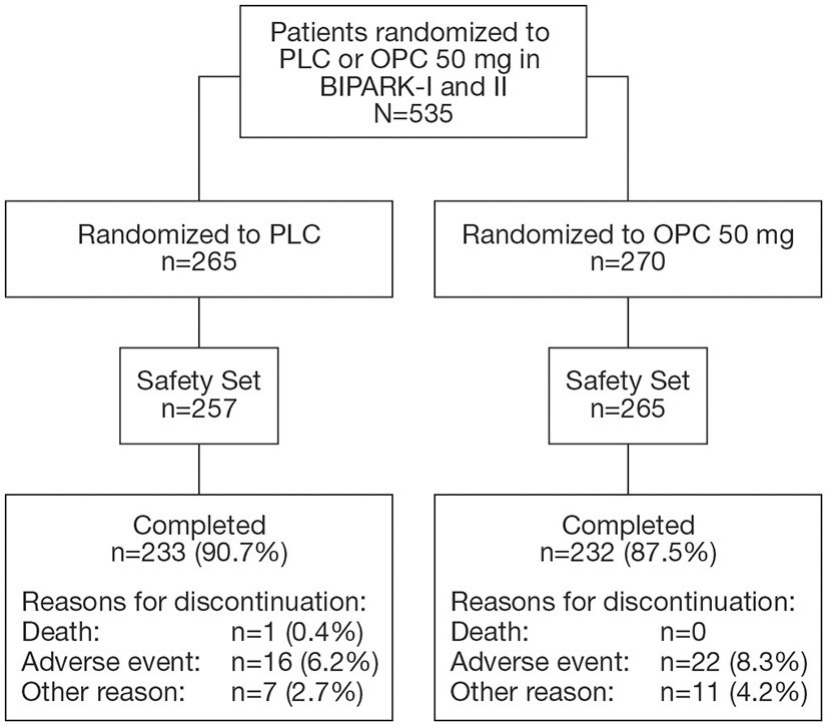
Flowchart of patient disposition. OPC, opicapone; PLC, placebo.

Five hundred and thirty-five patients were randomized to receive PLC or OPC 50 mg in BIPARK-I and II (Figure 1). The Safety Set included 522 patients (PLC, *n* = 257; OPC 50 mg, *n* = 265). In the overall OPC 50 mg Safety Set, 60.4% of patients were male, mean (SD) age was 64.5 (8.8) years, mean (SD) duration of PD was 7.6 (4.3) years, mean (SD) time since onset of motor fluctuations was 2.7 (2.9) years, mean (SD) Hoehn and Yahr staging at ON was 2.4 (0.5), mean (SD) absolute OFF-time at baseline was 6.2 (2.0) h, mean (SD) L-DOPA dose at baseline was 698.4 (322.1) mg/day, and mean (SD) duration of L-DOPA therapy was 6.3 (4.4) years. Baseline characteristics of the overall PLC Safety Set were similar to the OPC 50 mg Safety Set (18). Baseline characteristics of the OPC 50 mg and PLC subgroups are summarized in Supplementary Tables 1, 2, respectively.

### Safety/tolerability

#### TEAEs

In patients treated with OPC 50 mg, the incidence of TEAEs was lower in the subgroups of patients who were in “earlier” vs. “later” stages of their disease course and L-DOPA treatment pathway for 19 of the 22 pairwise comparisons (Table 1). The three exceptions were: patients with Hoehn and Yahr staging <2.5 vs. ≥2.5 (66.4% vs. 62.5%); patients treated with L-DOPA only vs. those not treated with L-DOPA only (72.1% vs. 61.4%); and patients treated with L-DOPA without a DA vs. patients treated with L-DOPA plus a DA (74.1% vs. 59.4%). In patients treated with PLC, the incidence was lower in 14 of the 22 “earlier” vs. “later” pairwise comparisons, and the between-group differences between pairwise comparator subgroups were generally smaller than for the OPC 50 mg pairwise comparator subgroups (Supplementary Table 3).

**Table 1:** Summary of TEAEs, related^a^ TEAEs, related^a^ serious TEAEs, and related^a^ TEAEs leading to discontinuation in specific OPC 50 mg subgroups (Safety Set).

**Subgroup**		***N***	**Any TEAE, %**	**Any related^a^ TEAE, %**	**Any related^a^ serious TEAE, %**	**Any related^a^ TEAE leading to discontinuation, %**
**Disease related**						
*Duration of PD (years)*	* <6*	119	**59.7**	**34.5**	0.8	7.6
	*≥6*	146	67.8	49.3	**0.7**	**7.5**
	* <7*	146	**58.9**	**32.9**	**0.7**	**6.2**
	*≥7*	119	70.6	54.6	0.8	9.2
	* <8*	162	**59.9**	**34.0**	**0.6**	**5.6**
	*≥8*	103	70.9	56.3	1.0	10.7
	* <9*	182	**60.4**	**34.6**	**0.5**	**6.6**
	*≥9*	83	72.3	60.2	1.2	9.6
*Hoehn and Yahr staging*	* <2.5*	113	66.4	46.9	0.9	8.0
	*≥2.5*	152	**62.5**	**39.5**	**0.7**	**7.2**
*Onset of MF (years)*	* ≤ 1*	85	**61.2**	**35.3**	1.2	**5.9**
	*>1*	162	64.8	44.4	**0.6**	8.6
	* ≤ 2*	143	**59.4**	**32.9**	**0.7**	**4.9**
	*>2*	104	69.2	52.9	1.0	11.5
**Therapy related**						
*L-DOPA intakes (n)*	* <4*	60	**50.0**	**25.0**	**0**	8.3
	*≥4*	205	68.3	47.8	1.0	**7.3**
	* <5*	132	**55.3**	**27.3**	0.8	8.3
	*≥5*	133	72.9	57.9	0.8	**6.8**
	* <6*	205	**61.5**	**36.6**	**0.5**	8.8
	*≥6*	60	73.3	63.3	1.7	**3.3**
*L-DOPA duration (years)*	* <4*	97	**59.8**	**30.9**	1.0	**5.2**
	*≥4*	168	66.7	49.4	**0.6**	8.9
	* <5*	125	**61.6**	**35.2**	0.8	**4.8**
	*≥5*	140	66.4	49.3	**0.7**	10.0
	* <6*	151	**59.6**	**34.4**	**0.7**	**7.3**
	*≥6*	114	70.2	53.5	0.9	7.9
	* <7*	174	**59.8**	**35.1**	**0.6**	**6.3**
	*≥7*	91	72.5	57.1	1.1	9.9
	* <8*	190	**61.6**	**36.8**	**0.5**	**5.8**
	*≥8*	75	70.7	57.3	1.3	12.0
*L-DOPA daily amount (mg)*	* <500*	66	**59.1**	**30.3**	**0**	7.6
	*≥500*	199	65.8	46.7	1.0	**7.5**
	* <600*	103	**60.2**	**34.0**	**0**	**5.8**
	*≥600*	162	66.7	48.1	1.2	8.6
	* <700*	144	**58.3**	**33.3**	**0.7**	**6.3**
	*≥700*	121	71.1	53.7	0.8	9.1
	* <800*	176	**60.8**	**36.9**	**0.6**	**7.4**
	*≥800*	89	70.8	53.9	1.1	7.9
*Use of L-DOPA only*	*Yes*	68	72.1	44.1	1.5	**7.4**
	*No*	197	**61.4**	**42.1**	**0.5**	7.6
*Use of L-DOPA plus DA*	*Yes*	180	**59.4**	**39.4**	**0**	**6.7**
	*No*	85	74.1	49.4	2.4	9.4
*Use of L-DOPA plus MAO-BI*	*Yes*	57	70.2	56.1	1.8	10.5
	*No*	208	**62.5**	**38.9**	**0.5**	**6.7**

Rows shaded in gray indicate variables associated with earlier use of L-DOPA adjunctive therapy and earlier disease course, in comparison with matched unshaded rows. Values shown in bold indicate variables for which the incidence of TEAEs was lower than that of the matched comparative row.

a TEAEs for which the relationship to study drug was reported as “possible,” “probable,” “definite” or missing.

DA, dopamine agonist; L-DOPA, levodopa; MAO-BI, monoamine oxidase B inhibitor; MF, motor fluctuations; OPC, opicapone; PD, Parkinson's disease; TEAE, treatment-emergent adverse event.

#### Related TEAEs

In patients treated with OPC 50 mg, the incidence of related TEAEs was lower for 19 of the 22 “earlier” vs. “later” pairwise comparisons conducted, and the three exceptions were the same as for TEAEs: patients with Hoehn and Yahr staging <2.5 vs. ≥2.5 (46.9% vs. 39.5%); patients treated with L-DOPA only vs. those not treated with L-dopa only (44.1% vs. 42.1%); and patients treated with L-DOPA without a DA vs. patients treated with L-dopa plus a DA (56.1% vs. 38.9%) (Table 1). In patients treated with PLC, the incidence was lower in 17 of the 22 “earlier” vs. “later” pairwise comparisons, and, as with TEAEs, the between-group differences between pairwise comparator subgroups were generally smaller than for the OPC 50 mg pairwise comparator subgroups (Supplementary Table 3).

#### Related serious TEAEs

The incidence of related serious TEAEs was generally lower in patients treated with OPC 50 mg vs. PLC (Table 1; Supplementary Table 3). In patients treated with OPC 50 mg, the incidence of related serious TEAEs was lower for 14 of the 22 “earlier” vs. “later” pairwise comparisons conducted (Table 1). The exceptions were: patients with PD duration <6 vs. ≥6 years (0.8% vs. 0.7%); patients with Hoehn and Yahr staging <2.5 vs. ≥2.5 (0.9% vs. 0.7%); patients with L-DOPA treatment duration <4 vs. ≥4 years (1.0% vs. 0.6%) or <5 vs. ≥5 years (0.8% vs. 0.7%); patients treated with L-DOPA only vs. those not treated with L-dopa only (1.5% vs. 0.5%); and patients treated with L-DOPA without a DA vs. patients treated with L-dopa plus a DA (2.4% vs. 1.8%). For the comparison of patients who received <5 vs. ≥5 L-DOPA intakes, the incidence was the same (0.8%). In contrast, in patients treated with PLC, the incidence was lower in only 4 of the 22 “earlier” vs. “later” pairwise comparisons (Supplementary Table 3).

#### Related TEAEs leading to discontinuation

The incidence of related TEAEs leading to discontinuation was generally slightly higher in patients treated with OPC 50 mg vs. PLC (Table 1; Supplementary Table 3). In patients treated with OPC 50 mg, the incidence of related TEAEs leading to discontinuation was lower for 15 of the 22 “earlier” vs. “later” pairwise comparisons conducted (Table 1). The exceptions were: patients with PD duration <6 vs. ≥6 years (7.6% vs. 7.5%); patients with Hoehn and Yahr staging <2.5 vs. ≥2.5 (8.0% vs. 7.2%); patients who received <4 vs. ≥4 L-DOPA intakes (8.3% vs. 7.3%); patients who received <5 vs. ≥5 L-DOPA intakes (8.3% vs. 6.8%); patients who received <6 vs. ≥6 L-DOPA intakes (8.8% vs. 3.3%); patients who received an L-DOPA dose of <500 vs. ≥500 mg/day (7.6% vs. 7.5%); and patients treated with L-DOPA without a DA vs. patients treated with L-dopa plus a DA (9.4% vs. 6.7%). In patients treated with PLC, the incidence was lower in 10 of the 22 “earlier” vs. “later” pairwise comparisons, and, for one comparison, the incidence was the same in both subgroups (Supplementary Table 3).

#### Related dopaminergic-related TEAEs

In patients treated with OPC 50 mg, the incidence of related dyskinesia was substantially lower for 21 of the 22 “earlier” vs. “later” pairwise comparisons, the exception being patients with Hoehn and Yahr staging <2.5 vs. ≥2.5 (22.1% vs. 17.8%) (Table 2). The incidence of related dyskinesia leading to discontinuation was lower for 13 of the 22 comparisons, and the same for two of the comparisons. In patients treated with PLC, the incidence of related dyskinesia was lower than in patients treated with OPC 50 mg, and the incidence was lower for 20 of the 22 “earlier” vs. “later” pairwise comparisons conducted, although between-group differences between pairwise comparator subgroups were smaller than for the OPC 50 mg pairwise comparator subgroups (Supplementary Table 4). The incidence of related dyskinesia leading to discontinuation was lower for 21 of the 22 “earlier” vs. “later” PLC pairwise comparisons.

**Table 2:** Summary of related^a^ dopaminergic-related TEAEs and related^a^ dopaminergic-related TEAEs leading to discontinuation in specific OPC 50 mg subgroups (Safety Set).

**Subgroup**		***N***	**Related^a^** **dopaminergic-related TEAEs**									
			**Dyskinesia**		**Nausea**		**Hallucination^b^**		**Orthostatic hypotension**		**Vomiting**	
			**%**	**% leading to discontin** **uation**	**%**	**% leading to discontin** **uation**	**%**	**% leading to discontin** **uation**	**%**	**% leading to discontin** **uation**	**%**	**% leading** **to** **discontin** **uation**
**Disease related**												
*Duration of PD (years)*	* <6*	119	**5.9**	**2.5**	**2.5**	0.8	0.8	0	**0.8**	0	**0**	**0**
	*≥6*	146	30.8	3.4	2.7	**0.7**	**0.7**	0	1.4	0	2.7	2.1
	* <7*	146	**8.2**	**2.1**	**2.1**	**0.7**	**0.7**	0	**0.7**	0	**0**	**0**
	*≥7*	119	33.6	4.2	3.4	0.8	0.8	0	1.7	0	3.4	2.5
	* <8*	162	**10.5**	**1.9**	**1.9**	**0.6**	**0.6**	0	**0.6**	0	**0**	**0**
	*≥8*	103	34.0	4.9	3.9	1.0	1.0	0	1.9	0	3.9	2.9
	* <9*	182	**12.1**	**2.7**	**1.6**	**0.5**	**0.5**	0	**0.5**	0	**0.5**	**0.5**
	*≥9*	83	36.1	3.6	4.8	1.2	1.2	0	2.4	0	3.6	2.4
*Hoehn and Yahr staging*	* <2.5*	113	22.1	3.5	5.3	1.8	**0**	0	**0.9**	0	2.7	1.8
	*≥2.5*	152	**17.8**	**2.6**	**0.7**	**0**	1.3	0	1.3	0	**0.7**	**0.7**
*Onset of MF (years)*	* ≤ 1*	85	**11.8**	3.5	3.5	1.2	**0**	0	1.2	0	**0**	**0**
	*>1*	162	23.5	**3.1**	**2.5**	**0.6**	1.2	0	1.2	0	2.5	1.9
	* ≤ 2*	143	**11.2**	**2.1**	**2.1**	**0.7**	**0.7**	0	**0.7**	0	**0**	**0**
	*>2*	104	30.8	4.8	3.8	1.0	1.0	0	1.9	0	3.8	2.9
**Therapy related**												
*L-DOPA intakes (n)*	* <4*	60	**6.7**	3.3	**0**	**0**	**0**	0	**0**	0	**0**	**0**
	*≥4*	205	23.4	**2.9**	3.4	1.0	1.0	0	1.5	0	2.0	1.5
	* <5*	132	**9.1**	3.0	**1.5**	0.8	**0**	0	**0.8**	0	**0.8**	**0.8**
	*≥5*	133	30.1	3.0	3.8	0.8	1.5	0	1.5	0	2.3	1.5
	* <6*	205	**14.1**	3.9	3.4	1.0	**0.5**	0	**1.0**	0	**1.5**	**1.0**
	*≥6*	60	38.3	**0**	**0**	**0**	1.7	0	1.7	0	1.7	1.7
*L-DOPA duration (years)*	* <4*	97	**9.3**	3.1	3.1	1.0	1.0	0	**1.0**	0	**1.0**	**0**
	*≥4*	168	25.6	**3.0**	**2.4**	**0.6**	**0.6**	0	1.8	0	1.8	1.8
	* <5*	125	**8.8**	**2.4**	3.2	0.8	1.6	0	**0.8**	0	**0.8**	**0**
	*≥5*	140	29.3	3.6	**2.1**	**0.7**	**0**	0	1.4	0	2.1	2.1
	* <6*	151	**8.6**	3.3	2.6	**0.7**	1.3	0	**0.7**	0	**0.7**	**0**
	*≥6*	114	34.2	**2.6**	2.6	0.9	**0**	0	1.8	0	2.6	2.6
	* <7*	174	**10.9**	**2.9**	**2.3**	**0.6**	1.1	0	**0.6**	0	**0.6**	**0**
	*≥7*	91	36.3	3.3	3.3	1.1	**0**	0	2.2	0	3.3	3.3
	* <8*	190	**13.7**	**2.6**	2.1	**0.5**	1.1	0	**0.5**	0	**0.5**	**0**
	*≥8*	75	34.7	4.0	**0.4**	1.3	**0**	0	2.7	0	4.0	4.0
*L-DOPA daily amount (mg)*	* <500*	66	**9.1**	3.0	**1.5**	**0**	**0**	0	**0**	0	**0**	**0**
	*≥500*	199	23.1	3.0	3.0	1.0	1.0	0	1.5	0	2.0	1.5
	* <600*	103	**7.8**	**1.9**	**1.9**	**0**	**0**	0	**1.0**	0	**0**	**0**
	*≥600*	162	27.2	3.7	3.1	1.2	1.2	0	1.2	0	2.5	1.9
	* <700*	144	**9.7**	**2.8**	3.5	**0.7**	**0.7**	0	1.4	0	**0**	**0**
	*≥700*	121	31.4	3.3	**1.7**	0.8	0.8	0	**0.8**	0	3.3	2.5
	* <800*	176	**13.1**	**2.8**	2.8	**0.6**	**0.6**	0	1.1	0	**0.6**	**0.6**
	*≥800*	89	32.6	3.4	**2.2**	1.1	1.1	0	1.1	0	3.4	2.2
*Use of L-DOPA only*	*Yes*	68	**11.8**	**2.9**	**1.5**	**0**	1.5	0	**0**	0	**0**	**0**
	*No*	197	22.3	3.0	3.0	1.0	**0.5**	0	1.5	0	2.0	1.5
*Use of L-DOPA plus DA*	*Yes*	180	20.6	**1.7**	2.8	**0.6**	**0.6**	0	**1.1**	0	2.2	1.7
	*No*	85	**17.6**	5.9	**2.4**	1.2	1.2	0	1.2	0	**0**	**0**
*Use of L-DOPA plus MAO-BI*	*Yes*	57	28.1	5.3	5.3	1.8	**0**	0	1.8	0	**0**	**0**
	*No*	208	**17.3**	**2.4**	**1.9**	**0.5**	1.0	0	**1.0**	0	1.9	1.4

Rows shaded in grey indicate variables associated with earlier use of L-DOPA adjunctive therapy and earlier disease course, in comparison with matched unshaded rows. Values shown in bold indicate variables for which the incidence of TEAEs was lower than that of the matched comparative row.

a TEAEs for which the relationship to study drug was reported as “possible,” “probable,” “definite” or missing.

b For hallucination, the percentages shown are for the preferred term “Hallucination” and do not include the preferred terms “Hallucination, auditory,” “Hallucination, visual,” and “Hallucinations, mixed.”

DA, dopamine agonist; L-DOPA, levodopa; MAO-BI, monoamine oxidase B inhibitor; MF, motor fluctuations; OPC, opicapone; PD, Parkinson's disease; TEAE, treatment-emergent adverse event.

In patients treated with OPC 50 mg, the incidence of related nausea was lower for 13 of the 22 “earlier” vs. “later” pairwise comparisons (compared with 8/22 in patients treated with PLC); and the incidence of related nausea leading to discontinuation was lower for 14 of the 22 comparisons (whereas no patients treated with PLC discontinued due to related nausea) (Table 2; Supplementary Table 4). The incidence of related hallucination was lower for 13 of the 22 “earlier” vs. “later” pairwise comparisons in patients treated with OPC 50 mg (compared with 9/22 in patients treated with PLC); no patients treated with OPC 50 mg or PLC discontinued due to related hallucination. The incidence of related orthostatic hypotension was lower for 18 of the 22 “earlier” vs. “later” pairwise comparisons in patients treated with OPC 50 mg, and the incidence was the same for two comparisons; no patients treated with OPC 50 mg discontinued due to related orthostatic hypotension, and no patients treated with PLC experienced related orthostatic hypotension. The incidence of related vomiting was lower for 20 of the 22 “earlier” vs. “later” pairwise comparisons in patients treated with OPC 50 mg (compared with 16/22 in patients treated with PLC); similarly, the incidence of related vomiting leading to discontinuation was lower for 20 of the 22 OPC 50 mg comparisons (compared with 6/22 in patients treated with PLC) (Table 2; Supplementary Table 4).

## Discussion

In these exploratory *post-hoc* analyses, OPC 50 mg was shown to be generally well-tolerated overall, but better tolerated in patients who were earlier in their disease course and less advanced on their trajectory of L-DOPA dose requirements than in those who were in later stages. In patients treated with OPC 50 mg, the incidences of TEAEs, related TEAEs, related serious TEAEs, and related TEAEs leading to discontinuation were lower for patients in earlier vs. later stages of their disease course and L-DOPA treatment pathway in 86.4, 86.4, 63.6, and 68.2% of the 22 pairwise comparisons conducted, respectively. By comparison, in patients treated with PLC the corresponding percentages were 63.6, 77.3, 18.2, and 45.5%, respectively, and between-group differences were generally smaller than for the corresponding OPC 50 mg pairwise comparator subgroups.

Although tolerability appeared to be more favorable for most subgroup comparisons of patients who were in earlier vs. later stages of their L-DOPA treatment pathway, common exceptions were patients treated with L-DOPA only vs. those not treated with L-DOPA only, and patients treated with L-DOPA without a DA vs. patients treated with L-DOPA plus a DA. However, mean L-DOPA daily doses at baseline were higher in patients treated with L-DOPA only (730.3 mg) vs. those not treated with L-DOPA only (687.4 mg), and in patients treated with L-DOPA without a DA (717.8 mg) vs. patients treated with L-DOPA plus a DA (689.2 mg) (Supplementary Table 1), which may have influenced these findings. Another common exception was that tolerability appeared to be less favorable for most subgroup comparisons of patients with Hoehn and Yahr staging <2.5 vs. ≥2.5. Although the reasons for this are unclear, Hoehn and Yahr staging may be responsive to treatment effects and higher dopaminergic doses may therefore have induced lower Hoehn and Yahr staging, confounding the results.

In patients treated with OPC 50 mg, the incidences of related dopaminergic-related TEAEs (dyskinesia, nausea, hallucination, orthostatic hypotension and vomiting), and rates of discontinuation due to these related TEAEs, were lower for patients in earlier vs. later stages of their disease and L-DOPA treatment pathway in the majority of pairwise comparisons conducted. Between-group differences were particularly marked for the incidence of related dyskinesia, confirming previous evidence demonstrating a close association between disease duration and the occurrence of dyskinesias (19). As expected, the incidences of these specific related dopaminergic-related TEAEs were generally lower in patients treated with PLC than in those treated with OPC 50 mg. It should be noted that it was not possible to differentiate between peak dose and diphasic dyskinesia. However, as OPC does not cross the blood–brain barrier, the higher incidence of related dopaminergic-related TEAEs (particularly dyskinesia) observed with OPC 50 mg in comparison with PLC is likely to reflect the fact that OPC increases the bioavailability of L-DOPA (13). In patients treated with PLC, although the incidences were lower for patients in earlier vs. later stages of their disease course and L-DOPA treatment pathway in the majority of pairwise comparisons conducted, the between-group comparisons were smaller than for the corresponding OPC 50 mg between-group comparisons. Rates of discontinuation due to related dopaminergic-related TEAEs were also lower in patients treated with PLC vs. OPC 50 mg, and no PLC-treated patients discontinued due to related nausea, hallucination or orthostatic hypotension.

Using the same pairwise comparisons, we previously demonstrated that although OPC 50 mg is efficacious for all motor fluctuations, it appears to have enhanced efficacy in patients who are in earlier vs. later stages of their disease course and L-DOPA treatment pathway (17). The current study complements these findings by demonstrating that OPC 50 mg is also better tolerated when used to treat patients who are in earlier vs. later stages of their disease course and L-DOPA treatment pathway. The pathophysiological basis for this is unclear but may relate to less advanced nigrostriatal denervation, less severe pulsatile stimulation of the system and/or less extranigral pathology in early vs. later disease stages. Taken together, these findings indicate that patients at a relatively early stage of their disease course and treatment pathway may experience enhanced efficacy with OPC 50 mg without compromising safety/tolerability. The potential utility of OPC 50 mg in early PD is being investigated further in the EPSILON (**E**arly **P**arkin**S**on w**I**th **L**evodopa/DDC inhibitor and **O**picapo**N**e) study, a Phase III, double-blind, randomized, placebo-controlled, parallel-group trial that has been specifically designed to explore the potential of OPC to enhance the clinical benefit of levodopa in patients in the early stages of PD, without end-of-dose motor fluctuations (20).

It is important that antiparkinsonian treatments have safety/tolerability profiles that are acceptable to patients over the long term, thereby optimizing treatment compliance. OPC was developed with the aim of overcoming some of the safety/tolerability concerns associated with other COMT inhibitors used in the treatment of PD. Tolcapone has been associated with an increased risk of fulminant liver injury during post-marketing surveillance, and necessitates regular liver function monitoring (21). Both tolcapone and entacapone may cause diarrhea, and metabolites of tolcapone and entacapone may cause discoloration of the urine (22, 23). OPC is less likely to cause diarrhea and urine discoloration than tolcapone and entacapone, does not require liver enzyme monitoring, and, most importantly, has the advantage of being administered once daily (16, 24). Once-daily antiparkinsonian medications are associated with significantly better treatment compliance when compared with agents prescribed more frequently (25).

The current study was limited in being an exploratory *post-hoc* analysis but additional studies targeting specific patients classes are ongoing. The study was also limited by the small size of some of the subgroups, and by the interdependence of variables (such as disease duration and L-DOPA dose, fluctuation history and Hoehn and Yahr staging), which may have confounded the results. Nevertheless, its results indicate that OPC 50 mg is generally well-tolerated when used to treat patients with PD who have end-of-dose fluctuations, with an even more favorable tolerability profile in patients who are earlier, as opposed to later, in their disease course and L-DOPA treatment pathway.

## Data availability statement

The raw data supporting the conclusions of this article will be made available by the authors, without undue reservation.

## Ethics statement

The studies involving human participants were reviewed and approved by Institutional Review Boards at the participating sites. The patients/participants provided their written informed consent to participate in this study.

## Author contributions

All authors listed have made a substantial, direct, and intellectual contribution to the work and approved it for publication.

## Funding

The authors declare that this study received funding from BIAL-Portela & Ca, S.A. The funder was not involved in the study design, collection, analysis, interpretation of data, the writing of this article or the decision to submit it for publication. Editorial assistance was provided by John Scopes of m*X*m Medical Communications and funded by Bial-Portela & Ca, S.A.

## Conflict of interest

J-FR, DM, HG, and PS are employees of Bial-Portela & Cª, S.A. GE has received honoraria for advisory boards and consultancy from AbbVie Pharma, BIAL Pharma, Biogen GmbH, Desitin Pharma, STADA Pharma, NeuroDerm Inc.; speaker's honoraria from AbbVie Pharma, BIAL Pharma, Britannia Pharma, Desitin Pharma, Licher GmbH, UCB Pharma, Zambon Pharma; and royalties from Kohlhammer Verlag, Thieme Verlag. AL is funded by the Reta Lila Weston Institute of Neurological Studies, University College London, Institute of Neurology and reports consultancies from Britannia Pharmaceuticals and BIAL Portela. He also reports grants and/or research support from the Frances and Renee Hock Fund, and honoraria from Britannia Pharmaceuticals, BIAL, STADA, UCB, and Nordiclnfu Care. ET received honoraria for consultancy from TEVA, Bial, Prevail Therapeutics, Boehringer Ingelheim, Roche and BIOGEN, and has received funding for research from the Spanish Network for Research on Neurodegenerative Disorders (CIBERNED)-Instituto Carlos III (ISCIII), and The Michael J. Fox Foundation for Parkinson's Research (MJFF). JF has provided consultancy for Ipsen, GlaxoSmithKline, Novartis, Teva, Lundbeck, Solvay, Abbott, BIAL, Merck-Serono and Merz; and has received grants from GlaxoSmithKline, Grunenthal, Teva and Fundação MSD. WP has received lecture fees and honoraria for consultancy in relation to clinical drug development programs from Alterity, AbbVie, Affiris, AstraZeneca, Axovant, BIAL, Biogen, Britannia, Lilly, Lundbeck, NeuroDerm, Neurocrine, Denali Pharmaceuticals, Orion Pharma, Roche, Stada, Sunovion, Takeda, UCB and Zambon, as well as grant support from the MJFF and the EU FP7 & Horizon 2020 programs. OR has participated in advisory boards and/or provided consultancy for AbbVie, Adamas, Acorda, Addex, AlzProtect, ApoPharma, AstraZeneca, Axovant, Bial, Biogen, Britannia, Buckwang, CereSpir, Clevexel, Denali, INC Research, IPMDS, Lundbeck, Lupin, Merck, MundiPharma, NeurATRIS, NeuroDerm, Novartis, ONO Pharma, Osmotica, Parexel, Pfizer, Prexton Therapeutics, Quintiles, Roche, Sanofi, Servier, Sunovion, Theranexus, Takeda, Teva, UCB, Vectura, Watermark Research, XenoPort, XO, Zambon; received grants from Agence Nationale de la Recherche (ANR), CHU de Toulouse, France-Parkinson, INSERM-DHOS Recherche Clinique Translationnelle, MJFox Foundation, Programme Hospitalier de Recherche Clinique, European Commission (FP7, H2020), Cure Parkinson UK; and received a grant to participate in a symposium and contribute to the review of an article by the International Parkinson and Movement Disorder Society. FS has received compensation for consultancy and speaker-related activities from Lundbeck, UCB, Chiesi, Zambon, Britannia, Cynapsus, Sunovion, Kyowa, Abbvie, Neuroderm, Biogen, Bial. AA has received compensation for consultancy and speaker-related activities from UCB, Boehringer Ingelheim, Britannia, AbbVie, Zambon, Bial, NeuroDerm, Theravance Biopharma, Roche; he receives research support from Chiesi Pharmaceuticals, Lundbeck, Horizon 2020 - Grant 825785, Horizon2020 Grant 101016902, Ministry of Education University and Research (MIUR) Grant ARS01_01081, Cariparo Foundation. He serves as consultant for Boehringer Ingelheim for legal cases on pathological gambling; owns Patent WO2015110261-A1; and owns shares in PD Neurotechnology Limited.

## Publisher's note

All claims expressed in this article are solely those of the authors and do not necessarily represent those of their affiliated organizations, or those of the publisher, the editors and the reviewers. Any product that may be evaluated in this article, or claim that may be made by its manufacturer, is not guaranteed or endorsed by the publisher.
