# Supplementary material for: The safety/tolerability of opicapone when used early in Parkinson's disease patients with levodopa-induced motor fluctuations: A post-hoc analysis of BIPARK-I and II
Source: Front Neurol. 2022 Aug 23;13:994114. doi: 10.3389/fneur.2022.994114 (PMC9446144; doi:10.3389/fneur.2022.994114)
Supplement: Supplementary file 1 [file Data_Sheet_1.docx]

**The safety/tolerability of opicapone when used early in Parkinson’s disease patients with levodopa-induced motor fluctuations: a *post-hoc* analysis of BIPARK-I and II**

**José‐Francisco Rocha,^1^* Georg Ebersbach,^2^ Andrew Lees,^3^ Eduardo Tolosa,^4^ Joaquim J. Ferreira,^5^ Werner Poewe,^6^ Olivier Rascol,^7^ Fabrizio Stocchi,^8^ Angelo Antonini,^9^ Diogo Magalhães,^1^ Helena Gama,^1^ Patrício Soares-da-Silva^1^**

^1^BIAL – Portela & C^a^ S.A., Coronado, Portugal; ^2^Movement Disorders Clinic, Beelitz-Heilstätten, Germany; ^3^National Hospital for Neurology and Neurosurgery, London, UK; ^4^Parkinson Disease and Movement Disorder Unit, Neurology Service, Hospital Clínic de Barcelona, Institut d'Investigacions Biomèdiques August Pi i Sunyer (IDIBAPS), University of Barcelona (UB), Centro de Investigación Biomédica en Red sobre Enfermedades Neurodegenerativas (CIBERNED) Barcelona, Spain; ^5^Laboratory of Clinical Pharmacology and Therapeutics, Faculty of Medicine, University of Lisbon, Lisbon, Portugal; ^6^Department of Neurology, Medical University of Innsbruck, Innsbruck, Austria; ^7^Toulouse Parkinson Expert Center, Departments of Neurosciences and Clinical Pharmacology, Centre d’Investigation Clinique de Toulouse CIC1436, NS-Park/FCRIN Network, and NeuroToul COEN Center, University Hospital of Toulouse, INSERM, University of Toulouse 3, Toulouse, France; ^8^Department of Neurology, IRCCS San Raffaele Pisana, Rome, Italy; ^9^Parkinson and Movement Disorders Unit, Center for Neurodegenerative disease (CESNE), Department of Neurosciences, University of Padova, Padova, Italy

***Corresponding author:**

José‐Francisco Rocha

BIAL – Portela & Ca S.A.,

Coronado,

Portugal

Tel: +351 22 986 6100

Email: [francisco.rocha@bial.com](mailto:francisco.rocha@bial.com)

**Supplementary Table 1. Baseline characteristics of OPC 50 mg patient subgroups (Safety Set)**

| **Subgroup** | | **N** | **Baseline** | | | | | | | |
| --- | --- | --- | --- | --- | --- | --- | --- | --- | --- | --- |
|  |  |  | **Age (mean [SD] years)** | **Duration of PD (mean [SD] years)** | **Absolute OFF-time (mean [SD] h)** | **Time since onset of motor fluctuations (mean [SD] years)** | **Hoehn and Yahr staging at ON (mean [SD])** | **Male sex (n [%])** | **L-DOPA dose (mean [SD] mg)** | **Duration of L-DOPA therapy (mean [SD] years)** |
| **Disease-related subgroups** | | | | | | | | | | |
| *Duration of PD (years)* | *<6* | 119 | 63.8 (9.5) | 4.3 (0.9) | 6.3 (1.8) | 1.4 (1.1) | 2.4 (0.6) | 81 (68.1) | 577.8 (301.8) | 3.5 (1.4) |
|  | *≥6* | 146 | 65.1 (8.3) | 10.4 (4.0) | 6.2 (2.2) | 3.9 (3.4) | 2.4 (0.5) | 79 (54.1) | 796.6 (305.2) | 8.6 (4.6) |
|  | *<7* | 146 | 63.9 (9.4) | 4.7 (1.2) | 6.2 (1.9) | 1.5 (1.1) | 2.4 (0.6) | 97 (66.4) | 615.1 (311.5) | 3.7 (1.6) |
|  | *≥7* | 119 | 65.3 (8.0) | 11.3 (3.9) | 6.3 (2.2) | 4.3 (3.6) | 2.5 (0.5) | 63 (52.9) | 800.6 (306.2) | 9.5 (4.6) |
|  | *<8* | 162 | 64.0 (9.2) | 5.0 (1.4) | 6.3 (2.0) | 1.6 (1.3) | 2.4 (0.6) | 105 (64.8) | 625.2 (306.4) | 3.9 (1.8) |
|  | *≥8* | 103 | 65.4 (8.1) | 11.9 (3.9) | 6.2 (2.1) | 4.5 (3.7) | 2.4 (0.5) | 55 (53.4) | 813.5 (313.9) | 10.1 (4.5) |
|  | *<9* | 182 | 64.2 (9.2) | 5.4 (1.7) | 6.3 (2.0) | 1.8 (1.5) | 2.4 (0.6) | 116 (63.7) | 652.5 (326.7) | 4.3 (2.1) |
|  | *≥9* | 83 | 65.1 (8.0) | 12.7 (3.9) | 6.0 (2.1) | 4.9 (3.9) | 2.4 (0.5) | 44 (53.0) | 799.1 (289.2) | 10.8 (4.8) |
| *Hoehn and Yahr staging* | *<2.5* | 113 | 63.0 (9.2) | 7.3 (4.3) | 6.1 (2.0) | 2.9 (3.1) | 1.9 (0.3) | 74 (65.5) | 678.3 (325.0) | 6.1 (4.3) |
|  | *≥2.5* | 152 | 65.6 (8.4) | 7.9 (4.3) | 6.4 (2.1) | 2.6 (2.7) | 2.8 (0.3) | 86 (56.6) | 713.3 (320.2) | 6.5 (4.4) |
| *Onset of MF (years)* | *≤1* | 85 | 63.7 (9.4) | 5.9 (2.8) | 6.0 (1.7) | 0.6 (0.3) | 2.4 (0.5) | 53 (62.4) | 616.6 (301.5) | 4.2 (2.9) |
|  | *>1* | 162 | 65.1 (8.6) | 8.6 (4.7) | 6.4 (2.1) | 3.8 (3.0) | 2.4 (0.5) | 98 (60.5) | 739.3 (323.3) | 7.4 (4.8) |
|  | *≤2* | 143 | 64.4 (9.1) | 6.0 (2.8) | 6.3 (2.0) | 1.0 (0.6) | 2.4 (0.5) | 92 (64.3) | 637.8 (316.0) | 4.5 (3.0) |
|  | *>2* | 104 | 64.9 (8.6) | 10.0 (4.9) | 6.3 (2.1) | 5.1 (3.1) | 2.4 (0.5) | 59 (56.7) | 778.6 (310.4) | 8.8 (5.0) |
| **Therapy-related subgroups** | | | | | | | | | | |
| *L-DOPA intakes (n)* | *<4* | 60 | 65.5 (9.4) | 5.6 (2.6) | 6.0 (1.7) | 1.7 (1.6) | 2.3 (0.6) | 40 (66.7) | 488.5 (198.4) | 4.3 (2.7) |
|  | *≥4* | 205 | 64.2 (8.7) | 8.2 (4.5) | 6.3 (2.1) | 3.1 (3.1) | 2.5 (0.5) | 120 (58.5) | 759.8 (325.8) | 6.9 (4.6) |
|  | *<5* | 132 | 65.4 (9.1) | 6.0 (3.0) | 6.0 (1.9) | 2.0 (1.9) | 2.4 (0.6) | 84 (63.6) | 560.4 (250.8) | 4.6 (3.0) |
|  | *≥5* | 133 | 63.6 (8.5) | 9.3 (4.7) | 6.5 (2.2) | 3.6 (3.5) | 2.4 (0.5) | 76 (57.1) | 835.3 (327.4) | 8.0 (4.9) |
|  | *<6* | 205 | 64.6 (9.1) | 6.9 (3.8) | 6.1 (2.0) | 2.4 (2.7) | 2.4 (0.5) | 127 (62.0) | 634.4 (285.3) | 5.6 (3.9) |
|  | *≥6* | 60 | 64.2 (8.0) | 10.1 (4.9) | 6.9 (2.2) | 3.9 (3.3) | 2.5 (0.5) | 33 (55.0) | 917.1 (346.4) | 8.8 (5.1) |
| *L-DOPA duration (years)* | *<4* | 97 | 63.5 (9.7) | 4.7 (1.8) | 6.2 (1.9) | 1.2 (0.9) | 2.4 (0.5) | 62 (63.9) | 592.4 (300.2) | 2.6 (0.8) |
|  | *≥4* | 168 | 65.1 (8.2) | 9.3 (4.4) | 6.3 (2.1) | 3.7 (3.2) | 2.4 (0.5) | 98 (58.3) | 759.6 (319.3) | 8.4 (4.2) |
|  | *<5* | 125 | 63.7 (9.5) | 4.9 (1.8) | 6.2 (1.9) | 1.3 (1.0) | 2.4 (0.5) | 79(63.2) | 587.5 (310.0) | 3.0 (1.1) |
|  | *≥5* | 140 | 65.3 (8.1) | 10.1 (4.3) | 6.3 (2.2) | 4.0 (3.6) | 2.4 (0.5) | 81 (57.9) | 797.4 (300.7) | 9.2 (4.1) |
|  | *<6* | 151 | 64.0 (9.4) | 5.1 (1.9) | 6.2 (1.8) | 1.5 (1.1) | 2.4 (0.5) | 95 (62.9) | 612.5 (319.0) | 3.4 (1.4) |
|  | *≥6* | 114 | 65.2 (8.1) | 11.0 (4.3) | 6.2 (2.3) | 4.4 (3.6) | 2.4 (0.5) | 65 (57.0) | 812.2 (290.5) | 10.1 (4.1) |
|  | *<7* | 174 | 64.1 (9.2) | 5.4 (2.0) | 6.2 (2.0) | 1.6 (1.2) | 2.4 (0.5) | 108 (62.1) | 631.5 (316.8) | 3.8 (1.6) |
|  | *≥7* | 91 | 65.3 (8.0) | 11.9 (4.2) | 6.2 (2.1) | 4.9 (3.8) | 2.5 (0.5) | 52 (57.1) | 826.3 (293.8) | 11.0 (4.1) |
|  | *<8* | 190 | 64.2 (9.1) | 5.7 (2.2) | 6.3 (2.1) | 1.7 (1.4) | 2.4 (0.5) | 117 (61.6) | 649.8 (314.0) | 4.1 (1.8) |
|  | *≥8* | 75 | 65.3 (8.0) | 12.5 (4.3) | 6.0 (2.0) | 5.2 (3.9) | 2.4 (0.6) | 43 (57.3) | 821.4 (311.6) | 11.8 (4.1) |
| *L-DOPA daily amount (mg)* | *<500* | 66 | 64.4 (10.1) | 6.0 (3.5) | 5.9 (2.0) | 2.1 (2.5) | 2.4 (0.6) | 39 (59.1) | 345.8 (78.8) | 4.6 (3.7) |
|  | *≥500* | 199 | 64.6 (8.4) | 8.2 (4.4) | 6.4 (2.1) | 3.0 (3.0) | 2.4 (0.5) | 121 (60.8) | 815.3 (284.8) | 6.9 (4.4) |
|  | *<600* | 103 | 64.7 (9.3) | 5.9 (3.0) | 5.8 (1.9) | 1.9 (2.2) | 2.4 (0.6) | 61 (59.2) | 404.0 (101.0) | 4.5 (3.2) |
|  | *≥600* | 162 | 64.4 (8.5) | 8.8 (4.6) | 6.5 (2.1) | 3.3 (3.1) | 2.4 (0.5) | 99 (61.1) | 885.6 (270.2) | 7.4 (4.6) |
|  | *<700* | 144 | 64.6 (9.1) | 6.5 (3.4) | 6.0 (2.0) | 2.1 (2.3) | 2.4 (0.6) | 80 (55.6) | 462.5 (126.6) | 5.1 (3.4) |
|  | *≥700* | 121 | 64.4 (8.5) | 9.0 (4.8) | 6.5 (2.1) | 3.5 (3.3) | 2.4 (0.5) | 80 (66.1) | 979.1 (250.9) | 7.8 (4.9) |
|  | *<800* | 176 | 64.4 (9.2) | 6.8 (3.7) | 6.1 (1.9) | 2.3 (2.6) | 2.4 (0.5) | 100 (56.8) | 512.6 (156.8) | 5.4 (3.7) |
|  | *≥800* | 89 | 64.7 (8.1) | 9.4 (4.8) | 6.6 (2.2) | 3.6 (3.3) | 2.4 (0.5) | 60 (67.4) | 1065.7 (238.7) | 8.1 (5.0) |
| *Use of L-DOPA only* | *Yes* | 68 | 65.6 (9.2) | 7.0 (4.3) | 6.6 (2.3) | 2.4 (2.6) | 2.5 (0.5) | 42 (61.8) | 730.3 (347.0) | 6.1 (4.4) |
|  | *No* | 197 | 64.2 (8.7) | 7.9 (4.3) | 6.1 (1.9) | 2.9 (3.0) | 2.4 (0.5) | 118 (59.9) | 687.4 (313.3) | 6.4 (4.4) |
| *Use of L-DOPA plus DA* | *Yes* | 180 | 64.5 (8.5) | 8.0 (4.3) | 6.1 (2.0) | 2.9 (3.0) | 2.4 (0.5) | 105 (58.3) | 689.2 (314.1) | 6.6 (4.4) |
|  | *No* | 85 | 64.6 (9.6) | 6.9 (4.1) | 6.5 (2.2) | 2.3 (2.5) | 2.4 (0.5) | 55 (64.7) | 717.8 (339.6) | 5.8 (4.2) |
| *Use of L-DOPA plus MAO-BI* | *Yes* | 57 | 62.8 (9.9) | 7.0 (4.0) | 5.9 (1.8) | 2.8 (3.0) | 2.3 (0.6) | 40 (70.2) | 604.2 (290.9) | 5.5 (4.2) |
|  | *No* | 208 | 65.0 (8.5) | 7.8 (4.3) | 6.3 (2.1) | 2.7 (2.8) | 2.5 (0.5) | 120 (57.7) | 724.2 (326.1) | 6.5 (4.4) |

Rows shaded in grey indicate variables generally associated with earlier disease course (shorter PD duration, lower Hoehn and Yahr staging, and shorter onset of MF; lower number of L-DOPA intakes, shorter duration of L-DOPA use, lower daily L-DOPA dose amount, and less use of adjunctive therapies), in comparison with matched unshaded rows. DA, dopamine agonist; L-DOPA, levodopa; MAO-BI, monoamine oxidase-B inhibitor; MF, motor fluctuations; OPC, opicapone; PD, Parkinson’s disease; SD, standard deviation.

**Supplementary Table 2. Baseline characteristics of PLC patient subgroups (Safety Set)**

| **Subgroup** | | **N** | **Baseline** | | | | | | | |
| --- | --- | --- | --- | --- | --- | --- | --- | --- | --- | --- |
|  |  |  | **Age (mean [SD] years)** | **Duration of PD (mean [SD] years)** | **Absolute OFF-time (mean [SD] h)** | **Time since onset of motor fluctuations (mean [SD] years)** | **Hoehn and Yahr staging at ON (mean [SD])** | **Male sex (n [%])** | **L-DOPA dose (mean [SD] mg)** | **Duration of L-DOPA therapy (mean [SD] years)** |
| **Disease-related subgroups** | | | | | | | | | | |
| *Duration of PD (years)* | *<6* | 103 | 63.3 (9.6) | 4.4 (0.9) | 6.1 (1.7) | 1.4 (1.1) | 2.3 (0.6) | 54 (52.4) | 542.4 (210.1) | 3.5 (1.4) |
|  | *≥6* | 154 | 62.6 (8.8) | 9.9 (3.6) | 6.2 (2.3) | 3.4 (2.3) | 2.4 (0.5) | 88 (57.1) | 796.2 (341.4) | 8.2 (3.5) |
|  | *<7* | 135 | 62.9 (9.2) | 4.9 (1.2) | 6.2 (1.8) | 1.6 (1.2) | 2.3 (0.6) | 70 (51.9) | 585.6 (267.1) | 3.9 (1.6) |
|  | *≥7* | 122 | 62.7 (9.1) | 10.9 (3.4) | 6.0 (2.3) | 3.7 (2.4) | 2.4 (0.5) | 72 (59.0) | 814.9 (332.6) | 9.0 (3.4) |
|  | *<8* | 156 | 62.9(9.2) | 5.2 (1.4) | 6.3 (1.9) | 1.7 (1.3) | 2.4 (0.6) | 84 (53.8) | 623.1 (316.3) | 4.2 (1.9) |
|  | *≥8* | 101 | 62.8 (9.1) | 11.6 (3.4) | 5.9 (2.4) | 3.9 (2.5) | 2.4 (0.5) | 58 (57.4) | 804.7 (296.3) | 9.5 (3.5) |
|  | *<9* | 181 | 62.7 (9.3) | 5.6 (1.7) | 6.4 (2.0) | 1.9 (1.4) | 2.4 (0.6) | 103 (56.9) | 658.8 (325.0) | 4.7 (2.1) |
|  | *≥9* | 76 | 63.1 (8.7) | 12.7 (3.2) | 5.6 (2.2) | 4.2(2.7) | 2.4 (0.5) | 39 (51.3) | 779.5 (294.9) | 10.2 (3.7) |
| *Hoehn and Yahr staging* | *<2.5* | 114 | 59.6 (9.7) | 7.1 (3.5) | 5.9 (1.8) | 2.4 (1.8) | 1.9 (0.3) | 62 (54.4) | 658.0 (329.7) | 6.0 (3.5) |
|  | *≥2.5* | 143 | 65.4 (7.7) | 8.2 (4.2) | 6.3 (2.3) | 2.8 (2.4) | 2.8 (0.3) | 80 (55.9) | 723.5 (311.3) | 6.6 (3.8) |
| *Onset of MF (years)* | *≤1* | 71 | 63.9 (9.4) | 5.8 (2.6) | 5.8 (1.8) | 0.6 (0.3) | 2.4 (0.5) | 37 (52.1) | 585.4 (274.6) | 4.4 (2.8) |
|  | *>1* | 174 | 62.3 (9.1) | 8.5 (4.1) | 6.3 (2.2) | 3.4 (2.1) | 2.4 (0.6) | 96 (55.2) | 742.3 (324.6) | 7.2 (3.6) |
|  | *≤2* | 125 | 64.0 (9.1) | 6.0 (2.6) | 6.2 (2.0) | 1.0 (0.6) | 2.3 (0.5) | 69 (55.2) | 637.9 (300.3) | 4.8 (2.7) |
|  | *>2* | 120 | 61.5 (9.1) | 9.5 (4.3) | 6.2 (2.2) | 4.3 (1.9) | 2.4 (0.6) | 64 (53.3) | 758.2 (326.4) | 8.0 (3.8) |
| **Therapy-related subgroups** | | | | | | | | | | |
| *L-DOPA intakes (n)* | *<4* | 51 | 65.5 (9.0) | 5.8 (2.7) | 6.1 (2.0) | 1.7 (1.7) | 2.3 (0.6) | 26 (51.0) | 468.9 (200.2) | 4.5 (2.9) |
|  | *≥4* | 206 | 62.2 (9.1) | 8.2 (4.0) | 6.2 (2.1) | 2.8 (2.2) | 2.4 (0.6) | 116 (56.3) | 750.3 (320.6) | 6.8 (3.7) |
|  | *<5* | 132 | 64.2 (10.0) | 6.6 (3.1) | 6.0 (2.1) | 2.0 (1.6) | 2.3 (0.6) | 71 (53.8) | 550.8 (227.9) | 5.4 (3.2) |
|  | *≥5* | 125 | 61.4 (8.1) | 8.9 (4.4) | 6.3 (2.0) | 3.3 (2.4) | 2.5 (0.6) | 71 (56.8) | 846.2 (334.8) | 7.3 (3.9) |
|  | *<6* | 197 | 63.3 (9.4) | 7.0 (3.6) | 6.1 (2.0) | 2.2(1.8) | 2.4 (0.6) | 105 (53.3) | 618.7 (256.2) | 5.6 (3.3) |
|  | *≥6* | 60 | 61.4 (8.1) | 9.9 (4.0) | 6.4 (2.2) | 4.0 (2.6) | 2.4 (0.6) | 37 (61.7) | 943.4 (381.8) | 8.5 (4.0) |
| *L-DOPA duration (years)* | *<4* | 78 | 62.6 (10.2) | 4.9 (2.8) | 5.8 (1.5) | 1.3 (1.0) | 2.3 (0.6) | 43 (55.1) | 517.6 (232.8) | 2.6 (0.9) |
|  | *≥4* | 179 | 62.9 (8.7) | 9.0 (3.7) | 6.3 (2.3) | 3.1 (2.3) | 2.4 (0.5) | 99 (55.3) | 771.5 (323.6) | 7.9 (3.2) |
|  | *<5* | 105 | 62.6 (9.7) | 5.2 (2.9) | 6.0 (1.7) | 1.4 (0.9) | 2.3 (0.6) | 58 (55.2) | 543.9 (237.7) | 3.1 (1.1) |
|  | *≥5* | 152 | 63.0 (8.8) | 9.4 (3.6) | 6.2 (2.3) | 3.4 (2.3) | 2.4 (0.5) | 84 (55.3) | 798.5 (329.7) | 8.5 (3.1) |
|  | *<6* | 142 | 62.8 (9.1) | 5.5 (2.6) | 6.2 (1.9) | 1.7 (1.2) | 2.4 (0.6) | 78 (54.9) | 582.2 (245.4) | 3.7 (1.4) |
|  | *≥6* | 115 | 62.8 (9.2) | 10.4 (3.5) | 6.1 (2.3) | 3.8 (2.5) | 2.4 (0.5) | 64 (55.7) | 833.1 (348.2) | 9.6 (2.9) |
|  | *<7* | 162 | 62.7 (9.0) | 5.7 (2.5) | 6.3 (2.0) | 1.8 (1.3) | 2.4 (0.6) | 86 (53.1) | 612.7 (290.0) | 4.0 (1.6) |
|  | *≥7* | 95 | 63.1 (9.4) | 11.2 (3.4) | 6.0 (2.3) | 3.9 (2.6) | 2.4 (0.5) | 56 (58.9) | 834.0 (323.6) | 10.2 (2.8) |
|  | *<8* | 181 | 62.6 (9.1) | 6.0 (2.7) | 6.2 (1.9) | 1.9 (1.4) | 2.4 (0.6) | 97 (53.6) | 642.9 (316.8) | 4.4 (1.8) |
|  | *≥8* | 76 | 63.4 (9.2) | 11.9 (3.2) | 5.9 (2.4) | 4.2 (2.7) | 2.4 (0.5) | 45 (59.2) | 817.3 (297.1) | 10.9 (2.6) |
| *L-DOPA daily amount (mg)* | *<500* | 69 | 64.0 (9.5) | 6.0 (2.7) | 6.0 (2.0) | 1.7 (1.5) | 2.2 (0.5) | 32 (46.4) | 353.1 (71.3) | 4.6 (3.1) |
|  | *≥500* | 188 | 62.4 (9.0) | 8.3 (4.1) | 6.2 (2.1) | 2.9 (2.3) | 2.4 (0.6) | 110 (58.5) | 819.8 (283.0) | 6.9 (3.7) |
|  | *<600* | 98 | 63.2 (9.4) | 6.1 (3.0) | 5.9 (2.0) | 2.0 (1.8) | 2.3 (0.6) | 45 (45.9) | 398.3 (92.7) | 4.7 (3.2) |
|  | *≥600* | 159 | 62.6 (9.0) | 8.7 (4.1) | 6.3 (2.1) | 3.0 (2.2) | 2.5 (0.5) | 97 (61.0) | 877.0 (270.8) | 7.3 (3.6) |
|  | *<700* | 139 | 63.3 (9.4) | 6.7 (3.5) | 6.1 (2.0) | 2.1 (1.9) | 2.3 (0.6) | 60 (43.2) | 462.6 (127.2) | 5.2 (3.2) |
|  | *≥700* | 118 | 62.2 (8.8) | 8.9 (4.1) | 6.2 (2.2) | 3.1 (2.3) | 2.4 (0.6) | 82 (69.5) | 967.6 (258.2) | 7.6 (3.8) |
|  | *<800* | 172 | 63.2 (9.4) | 7.0 (3.9) | 6.0 (2.0) | 2.3 (2.0) | 2.3 (0.6) | 80 (46.5) | 514.0 (156.1) | 5.4 (3.4) |
|  | *≥800* | 85 | 62.2 (8.7) | 9.1 (3.6) | 6.4 (2.3) | 3.3 (2.3) | 2.5 (0.6) | 62 (72.9) | 1059.7 (248.8) | 8.1 (3.6) |
| *Use of L-DOPA only* | *Yes* | 59 | 64.4 (9.8) | 6.2 (2.8) | 6.6 (2.3) | 1.8 (1.4) | 2.3 (0.6) | 36 (61.0) | 718.3 (359.1) | 5.1 (3.0) |
|  | *No* | 198 | 62.4 (8.9) | 8.2 (4.1) | 6.0 (2.0) | 2.9 (2.3) | 2.4 (0.6) | 106 (53.5) | 687.4 (308.8) | 6.7 (3.8) |
| *Use of L-DOPA plus DA* | *Yes* | 187 | 62.2 (8.8) | 8.1 (4.1) | 6.1 (2.0) | 2.9 (2.3) | 2.4 (0.6) | 98 (52.4) | 678.3 (304.7) | 6.6 (3.8) |
|  | *No* | 70 | 64.6 (9.7) | 6.5 (3.1) | 6.4 (2.2) | 1.9 (1.6) | 2.3 (0.6) | 44 (62.9) | 737.7 (358.5) | 5.4 (3.1) |
| *Use of L-DOPA plus MAO-BI* | *Yes* | 49 | 62.8 (9.0) | 8.3 (4.3) | 5.8 (2.0) | 2.4 (1.9) | 2.2 (0.6) | 32 (65.3) | 656.4 (325.2) | 6.0 (3.5) |
|  | *No* | 208 | 62.9 (9.2) | 7.6 (3.8) | 6.2 (2.1) | 2.6 (2.2) | 2.4 (0.5) | 110 (52.9) | 703.4 (319.6) | 6.4 (3.7) |

Rows shaded in grey indicate variables generally associated with earlier disease course (shorter PD duration, lower Hoehn and Yahr staging, and shorter onset of MF; lower number of L-DOPA intakes, shorter duration of L-DOPA use, lower daily L-DOPA dose amount, and less use of adjunctive therapies), in comparison with matched unshaded rows. DA, dopamine agonist; L-DOPA, levodopa; MAO-BI, monoamine oxidase-B inhibitor; MF, motor fluctuations; PD, Parkinson’s disease; PLC, placebo; SD, standard deviation.

**Supplementary Table 3. Summary of TEAEs, related^a^ TEAEs, related^a^ serious TEAEs, and related^a^ TEAEs leading to discontinuation in specific PLC subgroups (Safety Set)**

| **Subgroup** | | **N** | **Any TEAE, %** | **Any related^a^ TEAE, %** | **Any related^a^ serious TEAE, %** | **Any related^a^ TEAE leading to discontinuation, %** |
| --- | --- | --- | --- | --- | --- | --- |
|  |  |  |  |  |  |  |
| **Disease related** |  |  |  |  |  |  |
| *Duration of PD (years)* | *<6* | 103 | 57.3 | **28.2** | 2.9 | 5.8 |
|  | *≥6* | 154 | **57.1** | 29.9 | **1.9** | 5.8 |
|  | *<7* | 135 | **56.3** | **28.1** | 3.0 | 6.7 |
|  | *≥7* | 122 | 58.2 | 30.3 | **1.6** | **4.9** |
|  | *<8* | 156 | **57.1** | **28.2** | 3.2 | 7.7 |
|  | *≥8* | 101 | 57.4 | 30.7 | **1.0** | **3.0** |
|  | *<9* | 181 | **56.4** | **27.6** | 2.8 | 6.6 |
|  | *≥9* | 76 | 59.2 | 32.9 | **1.3** | **3.9** |
| *Hoehn and Yahr staging* | *<2.5* | 114 | 63.2 | 35.1 | 3.5 | 7.0 |
|  | *≥2.5* | 143 | **52.4** | **24.5** | **1.4** | **4.9** |
| *Onset of MF (years)* | *≤1* | 71 | **53.5** | **22.5** | 4.2 | **4.2** |
|  | *>1* | 174 | 56.9 | 31.6 | **1.7** | 6.3 |
|  | *≤2* | 125 | **55.2** | **24.8** | 3.2 | **4.0** |
|  | *>2* | 120 | 56.7 | 33.3 | **1.7** | 7.5 |
| **Therapy related** |  |  |  |  |  |  |
| *L-DOPA intakes (n)* | *<4* | 51 | **41.2** | **15.7** | **2.0** | **3.9** |
|  | *≥4* | 206 | 61.2 | 32.5 | 2.4 | 6.3 |
|  | *<5* | 132 | **56.1** | **28.0** | 3.0 | **4.5** |
|  | *≥5* | 125 | 58.4 | 30.4 | **1.6** | 7.2 |
|  | *<6* | 197 | **55.3** | **26.9** | 2.5 | **4.6** |
|  | *≥6* | 60 | 63.3 | 36.7 | **1.7** | 10.0 |
| *L-DOPA duration (years)* | *<4* | 78 | **55.1** | **25.6** | 2.6 | **5.1** |
|  | *≥4* | 179 | 58.1 | 30.7 | **2.2** | 6.1 |
|  | *<5* | 105 | 58.1 | **28.6** | 2.9 | **5.7** |
|  | *≥5* | 152 | **56.6** | 29.6 | **2.0** | 5.9 |
|  | *<6* | 142 | 59.2 | 31.0 | 2.8 | 7.7 |
|  | *≥6* | 115 | **54.8** | **27.0** | **1.7** | **3.5** |
|  | *<7* | 162 | 59.3 | 30.9 | 2.5 | 7.4 |
|  | *≥7* | 95 | **53.7** | **26.3** | **1.1** | **3.2** |
|  | *<8* | 181 | 58.6 | 29.3 | 2.8 | 7.2 |
|  | *≥8* | 76 | **53.9** | **28.9** | **1.3** | **2.6** |
| *L-DOPA daily amount (mg)* | *<500* | 69 | 58.0 | **26.1** | **1.4** | **5.8** |
|  | *≥500* | 188 | **56.9** | 30.3 | 2.7 | 5.9 |
|  | *<600* | 98 | **56.1** | **28.6** | **1.0** | 6.1 |
|  | *≥600* | 159 | 57.9 | 29.6 | 3.1 | **5.7** |
|  | *<700* | 139 | **55.4** | **26.6** | **2.2** | **5.8** |
|  | *≥700* | 118 | 59.3 | 32.2 | 2.5 | 5.9 |
|  | *<800* | 172 | **57.0** | 30.2 | 3.5 | 6.4 |
|  | *≥800* | 85 | 57.6 | **27.1** | **0** | **4.7** |
| *Use of L-DOPA only* | *Yes* | 59 | **55.9** | **22.0** | 3.4 | 8.5 |
|  | *No* | 198 | 57.6 | 31.3 | **2.0** | **5.1** |
| *Use of L-DOPA plus DA* | *Yes* | 187 | **56.1** | 31.0 | **2.1** | **4.8** |
|  | *No* | 70 | 60.0 | **24.3** | 2.9 | 8.6 |
| *Use of L-DOPA plus MAO-BI* | *Yes* | 49 | 71.4 | 38.8 | **0** | 6.1 |
|  | *No* | 208 | **53.8** | **26.9** | 2.9 | **5.8** |

Rows shaded in grey indicate variables associated with earlier use of L-DOPA adjunctive therapy and earlier disease course, in comparison with matched unshaded rows. Values shown in bold indicate variables for which the incidence of TEAEs was lower than that of the matched comparative row.

**^a^**TEAEs for which the relationship to study drug was reported as ‘possible’, ‘probable’, ‘definite’ or missing.

DA, dopamine agonist; L-DOPA, levodopa; MAO-BI, monoamine oxidase B inhibitor; MF, motor fluctuations; PD, Parkinson’s disease; PLC, placebo; TEAE, treatment-emergent adverse event.

**Supplementary Table 4. Summary of related^a^ dopaminergic-related TEAEs and related^a^ dopaminergic-related TEAEs leading to discontinuation in specific PLC subgroups (Safety Set)**

| **Subgroup** | | **N** | **Related^a^ dopaminergic-related TEAEs** | | | | | | | | | |
| --- | --- | --- | --- | --- | --- | --- | --- | --- | --- | --- | --- | --- |
|  |  |  | Dyskinesia | | Nausea | | Hallucination^b^ | | Orthostatic  hypotension | | Vomiting | |
|  |  |  | % | % leading to discontinuation | % | % leading to discontinuation | % | % leading to discontinuation | % | % leading to discontinuation | % | % leading to discontinuation |
| **Disease related** |  |  |  |  |  |  |  |  |  |  |  |  |
| *Duration of PD (years)* | *<6* | 103 | **3.9** | **0** | 3.9 | 0 | 1.0 | 0 | 0 | 0 | 1.0 | 1.0 |
|  | *≥6* | 154 | 7.8 | 0.6 | **2.6** | 0 | **0** | 0 | 0 | 0 | **0.6** | **0** |
|  | *<7* | 135 | **4.4** | **0** | 4.4 | 0 | 0.7 | 0 | 0 | 0 | **0.7** | 0.7 |
|  | *≥7* | 122 | 8.2 | 0.8 | **1.6** | 0 | **0** | 0 | 0 | 0 | 0.8 | **0** |
|  | *<8* | 156 | **3.8** | **0** | 3.8 | 0 | 0.6 | 0 | 0 | 0 | **0.6** | 0.6 |
|  | *≥8* | 101 | 9.9 | 1.0 | **2.0** | 0 | **0** | 0 | 0 | 0 | 1.0 | **0** |
|  | *<9* | 181 | **3.9** | **0** | 3.3 | 0 | 0.6 | 0 | 0 | 0 | **0.6** | 0.6 |
|  | *≥9* | 76 | 11.8 | 1.3 | **2.6** | 0 | **0** | 0 | 0 | 0 | 1.3 | **0** |
| *Hoehn and Yahr staging* | *<2.5* | 114 | 7.0 | **0** | 3.5 | 0 | **0** | 0 | 0 | 0 | 1.8 | 0.9 |
|  | *≥2.5* | 143 | **5.6** | 0.7 | **2.8** | 0 | 0.7 | 0 | 0 | 0 | **0** | **0** |
| *Onset of MF (years)* | *≤1* | 71 | **2.8** | **0** | **2.8** | 0 | 1.4 | 0 | 0 | 0 | **0** | 0 |
|  | *>1* | 174 | 8.0 | 0.6 | 3.4 | 0 | **0** | 0 | 0 | 0 | 0.6 | 0 |
|  | *≤2* | 125 | **3.2** | **0** | **3.2** | 0 | 0.8 | 0 | 0 | 0 | **0** | 0 |
|  | *>2* | 120 | 10.0 | 0.8 | 3.3 | 0 | **0** | 0 | 0 | 0 | 0.8 | 0 |
| **Therapy related** |  |  |  |  |  |  |  |  |  |  |  |  |
| *L-DOPA intakes (n)* | *<4* | 51 | **3.9** | **0** | **0** | 0 | **0** | 0 | 0 | 0 | **0** | **0** |
|  | *≥4* | 206 | 6.8 | 0.5 | 3.9 | 0 | 0.5 | 0 | 0 | 0 | 1.0 | 0.5 |
|  | *<5* | 132 | **4.5** | **0** | 3.8 | 0 | 0.8 | 0 | 0 | 0 | **0** | **0** |
|  | *≥5* | 125 | 8.0 | 0.8 | **2.4** | 0 | **0** | 0 | 0 | 0 | 1.6 | 0.8 |
|  | *<6* | 197 | **5.6** | **0** | **3.0** | 0 | 0.5 | 0 | 0 | 0 | **0** | **0** |
|  | *≥6* | 60 | 8.3 | 1.7 | 3.3 | 0 | **0** | 0 | 0 | 0 | 3.3 | 1.7 |
| *L-DOPA duration (years)* | *<4* | 78 | **2.6** | **0** | **1.3** | 0 | **0** | 0 | 0 | 0 | 1.3 | 1.3 |
|  | *≥4* | 179 | 7.8 | 0.6 | 3.9 | 0 | 0.6 | 0 | 0 | 0 | **0.6** | **0** |
|  | *<5* | 105 | **2.9** | **0** | **2.9** | 0 | 1.0 | 0 | 0 | 0 | 1.0 | 1.0 |
|  | *≥5* | 152 | 8.6 | 0.7 | 3.3 | 0 | **0** | 0 | 0 | 0 | **0.7** | **0** |
|  | *<6* | 142 | **4.2** | **0** | 3.5 | 0 | 0.7 | 0 | 0 | 0 | **0.7** | 0.7 |
|  | *≥6* | 115 | 8.7 | 0.9 | **2.6** | 0 | **0** | 0 | 0 | 0 | 0.9 | **0** |
|  | *<7* | 162 | **4.9** | **0** | 3.7 | 0 | 0.6 | 0 | 0 | 0 | **0.6** | 0.6 |
|  | *≥7* | 95 | 8.4 | 1.1 | **2.1** | 0 | **0** | 0 | 0 | 0 | 1.1 | **0** |
|  | *<8* | 181 | **4.4** | **0** | 3.3 | 0 | 0.6 | 0 | 0 | 0 | **0.6** | 0.6 |
|  | *≥8* | 76 | 10.5 | 1.3 | **2.6** | 0 | **0** | 0 | 0 | 0 | 1.3 | **0** |
| *L-DOPA daily amount (mg)* | *<500* | 69 | **2.9** | **0** | 4.3 | 0 | **0** | 0 | 0 | 0 | 1.4 | 0 |
|  | *≥500* | 188 | 7.4 | 0.5 | **2.7** | 0 | 0.5 | 0 | 0 | 0 | **0.5** | 0 |
|  | *<600* | 98 | **3.1** | **0** | 3.1 | 0 | **0** | 0 | 0 | 0 | 1.0 | 1.0 |
|  | *≥600* | 159 | 8.2 | 0.6 | 3.1 | 0 | 0.6 | 0 | 0 | 0 | **0.6** | **0** |
|  | *<700* | 139 | **2.9** | **0** | **2.2** | 0 | **0** | 0 | 0 | 0 | **0.7** | 0.7 |
|  | *≥700* | 118 | 10.2 | 0.8 | 4.2 | 0 | 0.8 | 0 | 0 | 0 | 0.8 | **0** |
|  | *<800* | 172 | **4.1** | **0** | **2.3** | 0 | 0.6 | 0 | 0 | 0 | **0.6** | 0.6 |
|  | *≥800* | 85 | 10.6 | 1.2 | 4.7 | 0 | **0** | 0 | 0 | 0 | 1.2 | **0** |
| *Use of L-DOPA only* | *Yes* | 59 | **1.7** | **0** | 6.8 | 0 | **0** | 0 | 0 | 0 | **0** | **0** |
|  | *No* | 198 | 7.6 | 0.5 | **2.0** | 0 | 0.5 | 0 | 0 | 0 | 1.0 | 0.5 |
| *Use of L-DOPA plus DA* | *Yes* | 187 | 7.5 | **0** | **2.1** | 0 | 0.5 | 0 | 0 | 0 | 1.1 | 0.5 |
|  | *No* | 70 | **2.9** | 1.4 | 5.7 | 0 | **0** | 0 | 0 | 0 | **0** | **0** |
| *Use of L-DOPA plus MAO-BI* | *Yes* | 49 | **4.1** | 2.0 | **2.0** | 0 | **0** | 0 | 0 | 0 | 2.0 | 2.0 |
|  | *No* | 208 | 6.7 | **0** | 3.4 | 0 | 0.5 | 0 | 0 | 0 | **0.5** | **0** |

Rows shaded in grey indicate variables associated with earlier use of L-DOPA adjunctive therapy and earlier disease course, in comparison with matched unshaded rows. Values shown in bold indicate variables for which the incidence of TEAEs was lower than that of the matched comparative row.

**^a^**TEAEs for which the relationship to study drug was reported as ‘possible’, ‘probable’, ‘definite’ or missing. ^b^For hallucination, the percentages shown are for the preferred term ‘Hallucination’ and do not include the preferred terms ‘Hallucination, auditory’, ‘Hallucination, visual’ and ‘Hallucinations, mixed’.

DA, dopamine agonist; L-DOPA, levodopa; MAO-BI, monoamine oxidase B inhibitor; MF, motor fluctuations; PD, Parkinson’s disease; PLC, placebo; TEAE, treatment-emergent adverse event.
